# Meroterpenoids from the Brown Alga *Cystoseira usneoides* as Potential Anti-Inflammatory and Lung Anticancer Agents

**DOI:** 10.3390/md18040207

**Published:** 2020-04-11

**Authors:** Hanaa Zbakh, Eva Zubía, Carolina de los Reyes, José M. Calderón-Montaño, Miguel López-Lázaro, Virginia Motilva

**Affiliations:** 1 Department of Pharmacology, Faculty of Pharmacy, University of Seville, Seville 41012, Spain; ha.zbakh@gmail.com (H.Z.); jcalderon@us.es (J.M.C.-M.); mlopezlazaro@us.es (M.L.-L.); 2 Department of Biology, Faculty of Sciences, University of Abdelmalek Essaâdi, Tetouan 93000, Morocco; 3 Department of Organic Chemistry, Faculty of Marine and Environmental Sciences, University of Cadiz, Puerto Real (Cádiz) 11510, Spain; eva.zubia@uca.es (E.Z.); carolina.dereyes@uca.es (C.d.l.R.)

**Keywords:** meroterpenoids, *Cystoseira usneoides*, inflammation, cytokines, lung cancer, cell cycle

## Abstract

The anti-inflammatory and anticancer properties of eight meroterpenoids isolated from the brown seaweed *Cystoseira usneoides* have been evaluated. The algal meroterpenoids (AMTs) **1–8** were tested for their inhibitory effects on the production of the pro-inflammatory cytokines tumor necrosis factor (TNF-α), interleukin-6 (IL-6), and interleukin-1β (IL-1β), and the expression of cyclooxygenase-2 (COX-2), and inducible nitric oxide synthase (iNOS) in LPS-stimulated THP-1 human macrophages. The anticancer effects were assessed by cytotoxicity assays against human lung adenocarcinoma A549 cells and normal lung fibroblastic MRC-5 cells, together with flow cytometry analysis of the effects of these AMTs on different phases of the cell cycle. The AMTs **1**–**8** significantly reduced the production of TNF-α, IL-6, and IL-1β, and suppressed the COX-2 and iNOS expression, in LPS-stimulated cells (*p* < 0.05). The AMTs **1**–**8** displayed higher cytotoxic activities against A549 cancer cells than against MRC-5 normal lung cells. Cell cycle analyses indicated that most of the AMTs caused the arrest of A549 cells at the G2/M and S phases. The AMTs 2 and 5 stand out by combining significant anti-inflammatory and anticancer activities, while 3 and 4 showed interesting selective anticancer effects. These findings suggest that the AMTs produced by *C. usneoides* may have therapeutic potential in inflammatory diseases and lung cancer.

## 1. Introduction

Inflammation is a physiologic process in response to invading pathogens or endogenous signals such as tissue injury. It is initiated by migration of immune cells from blood vessels and release of mediators, followed by recruitment of inflammatory cells and secretion of increased amounts of cytokines and chemokines to eliminate invading pathogens and to repair damaged tissues [1,2,3].

During the past decade, numerous epidemiological studies have consistently linked the immune system with tumorigenesis [4]. However, the role of inflammation in cancer is not new. Virchow postulated in 1863 that tumors arise in areas of chronic inflammation and that inflammation cells were present in the resected tumors [5,6]. These observations led him to hypothesize that inflammation is a predisposing factor of carcinogenesis [3,6]. It is now becoming clear that the immune system contributes to all stages of tumorigenesis, from initiation to invasion and metastasis of tumors, by providing abundant molecules to the tumor microenvironment. These molecules include growth factors, cytokines, and chemokines that increase mutagenesis, promote unregulated cell proliferation, limit apoptosis, and favor angiogenesis, invasion, and metastasis [4,7]. Nevertheless, the role of the immune system during carcinogenesis is complex, dynamic and ambivalent, and is now known to have the potential to both promote and revoke carcinogenic response [8,9,10].

Lung cancer causes 19% of all cancer deaths worldwide [11]; it is the most commonly diagnosed cancer and the leading cause of cancer-related mortality both in males and females [12,13]. The lung, as an organ of the respiratory system exposed to the outer environment, is a place predisposed for infections and chronic inflammatory injuries [14,15]. The chronic airway inflammation contributes to DNA damage, mutation, and pathological/molecular alterations in the bronchial epithelium and microenvironment, through the production of different cytokines, chemokines, and transcription factor networks, which increase lung tumor development and progression [14,15,16]. Hence, a strategy for the prevention and treatment of lung cancer could involve the regulation of inflammatory molecules, including pro-inflammatory cytokines, and inflammatory enzymes, such as cyclooxygenase-2 (COX-2) and inducible nitric oxide synthetase (iNOS) [17,18].

Since ancient times to nowadays, seaweeds have been used for food and diverse consumer products, due to their low content in lipids and high concentration in vitamins, minerals, proteins, dietary fiber, and polysaccharides [19,20,21]. In addition, a variety of metabolites from macroalgae have been shown to possess health promoting effects, including antioxidant [22], anti-inflammatory [23], antimicrobial [24], and antitumor [25] properties. Brown algae are a promising group of seaweeds known to be a rich source of bioactive compounds [26]. Among brown algae, the genus *Cystoseira*, which currently encompasses about 50 species distributed throughout the northeastern Atlantic Ocean and the Mediterranean Sea [27], has been widely studied, from both chemical and biological points of view [28]. In particular, many algae of the genus *Cystoseira* have been described to contain a variety of natural products of the meroditerpene class [28,29,30,31] some of which have been shown to possess anticancer and anti-inflammatory properties [28,29,32].

Hence, the current research has been aimed at expanding our investigation over the anti-inflammatory and anticancer effects of the algal meroterpenoids (AMTs) **1**–**8** previously isolated from the species *C. usneoides* [33]. Herein, we demonstrate that the AMTs **1**–**8** exhibit anti-inflammatory activities through the inhibition of pro-inflammatory cytokines (TNF-α, IL-6, and IL-1β), the protein expressions of COX-2 and iNOS in the LPS-stimulated THP-1 human macrophages, as well as that the AMTs **1**–**8** possess selective anticancer activity against human lung cancer cells A549 by inducing cell cycle arrest.

## 2. Results

**Figure 1:**
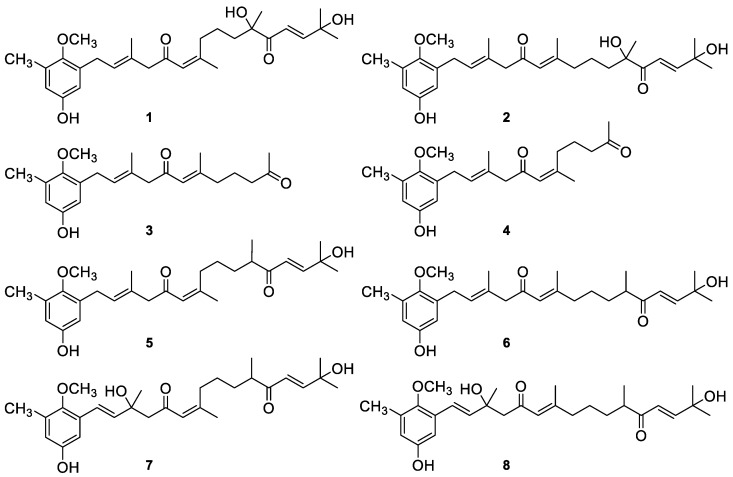
Chemical structures of the meroterpenes from C. usneoides subjected to anti-inflammatory and lung anticancer studies: usneoidone Z (**1**), 11-hydroxy-1′-*O*-methylamentadione (**2**), cystomexicone B (**3**), cystomexicone A (**4**), 6-*cis*-amentadione-1′-methyl ether (**5**), amentadione-1′-methyl ether (**6**), cystodione A (**7**), and cystodione B (**8**).

The algal meroterpenoids (AMTs) usneoidone Z (**1**), 11-hydroxy-1’-*O*-methylamentadione (**2**), cystomexicone B (**3**), cystomexicone A (**4**), 6-*cis*-amentadione-1′-methyl ether (**5**), amentadione-1′-methyl ether (**6**), cystodione A (**7**), and cystodione B (**8**) (Figure 1) isolated from the alga *C. usneoides* have been investigated for their anti-inflammatory and anticancer activities.

### 2.1. Anti-Inflammatory Activity

#### 2.1.1. Effects of AMTs **1**–**8** on the Viability of THP-1 Cells

The cytotoxic effect of AMTs **1**–**8** on LPS-stimulated THP-1 macrophages was determined at different concentrations and incubation times (0–100 µg/mL, 48 and 72 h) using the SRB assay. The results of this analysis demonstrated that none of the molecules affect cell viability at concentrations up to 10 µg/mL for AMTs **1**, **2**, **3**, **4**, **7**, **8**, and up to 6 µg/mL for AMTs **5** and **6** (data not shown). Therefore, in order to rule out cytotoxic effects, compounds **1**, **2**, **3**, **4**, **7**, and **8** were tested on THP-1 cells at maximum concentration of 8 µg/mL while **5** and **6** were tested at maximum concentration of 4 µg/mL.

#### 2.1.2. Effects of AMTs **1**–**8** on TNF-α, IL-6, and IL-1β Expression in LPS-stimulated THP-1 Macrophages

**Figure 2:**
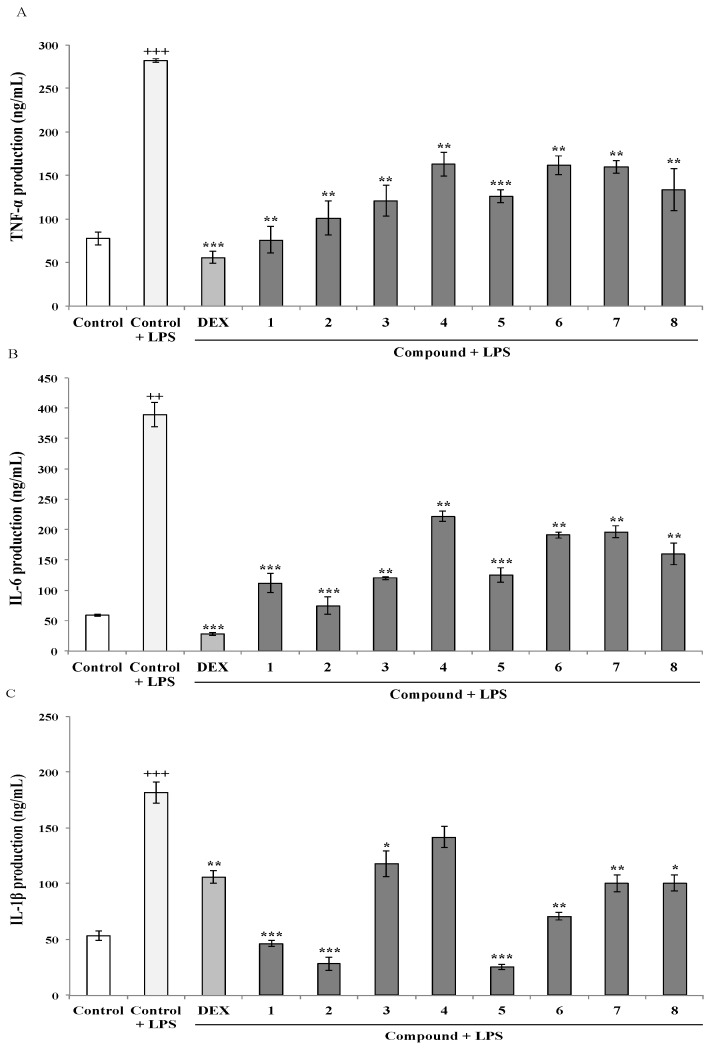
Meroterpenoids **1**–**8** inhibit LPS-induced expression of TNF-α, IL-6, and IL-1β in THP-1 macrophages (**A**, **B**, and **C**) respectively. Cells were pretreated for 1 h with the compounds (**1**, **2**, **3**, **4**, **7**, and **8** at 8 μg/mL; 5 and 6 at 4 μg/mL), followed by 24 h treatment with LPS. TNF-α (**A**), IL-6 (**B**) and IL-1β (**C**) contents in the culture medium were determined by ELISA. Dexametasone (DEX) was used as positive control. Data are expressed as means ± SE from three independent experiments. Statistical significance is indicated (+++*p* < 0.001 and +++< 0.01 vs. Control; respectively * *p* < 0.05, ** *p* < 0.01, *** *p* < 0.001 vs. Control + LPS).

To determine the effects of the AMTs **1**–**8** on the production of TNF-α, IL-6, and IL-1β, THP-1 macrophaghes were pretreated with the compounds and then stimulated with LPS, as the triggering factor to stimulate the cytokines production. The levels of pro-inflammatory cytokines in the cell supernatants were determined using the enzyme-linked immunosorbent (ELISA) kits. Upon comparison with the control cells, TNF-α, IL-6, and IL-1β levels were significantly increased in LPS stimulated cells up to 282.06, 389.47, and 181.80 ng/mL, respectively (Figure 2).

However, LPS-stimulated THP-1 macrophages pre-treated with the AMTs **1**–**8** showed a significant reduction of the production of pro-inflammatory cytokines (Figure 2). Regarding TNF-α, although all compounds induced a significant reduction of the level of this cytokine in THP-1 (Figure 2A), the meroditerpenes **1** and **2** showed the higher suppressive effect causing 73.11% and 64.14% inhibition. Compounds **3**, **5**, and **8** also induced more than 50% of inhibition (57.13%, 55.34%, and 52.56%, respectively), while compounds **4**, **6**, and **7** were less active, reducing the production of TNF-α between 42.18 and 43.32% (*p* < 0.01). As shown in Figure 2B, among the eight AMTs, compound **2** markedly inhibited LPS-induced IL-6 production in THP-1 macrophages by 80.81% and compounds **1**, **3**, and **5** caused strong inhibitions of 71.20%, 69.18% and 67.83%, respectively. The treatment of cells with compounds **4**, **6**, **7**, and **8** also significantly inhibited the production of IL-6 upon comparison with LPS-stimulated THP-1 control cells, although to a lesser extent (43.00%, 50.94%, 49.57% and 58.87%, respectively). With regard to IL-1β production, the pretreatment of cells with the AMTs **1**–**8** resulted in significant inhibition of this cytokine (Figure 2C). The most marked effects were observed in the cells treated with compounds **2** and **5**, which blocked the effect of 1 μg/mL LPS by 84.43% and 86.00%, respectively. Moreover, pretreatment with the AMTs **1** and **6** also strongly inhibited LPS-induced IL-1β production by 74.56% and 61.07%, respectively. The AMTs **3**, **7**, and **8** displayed more moderated inhibitory activity, causing IL-β decreases of 35.28%, 44.85%, and 44.60%, respectively.

#### 2.1.3. Effects of AMTs **1**–**8** on the Expression of COX-2 and iNOS Proteins in LPS-stimulated THP-1 Cells

**Figure 3:**
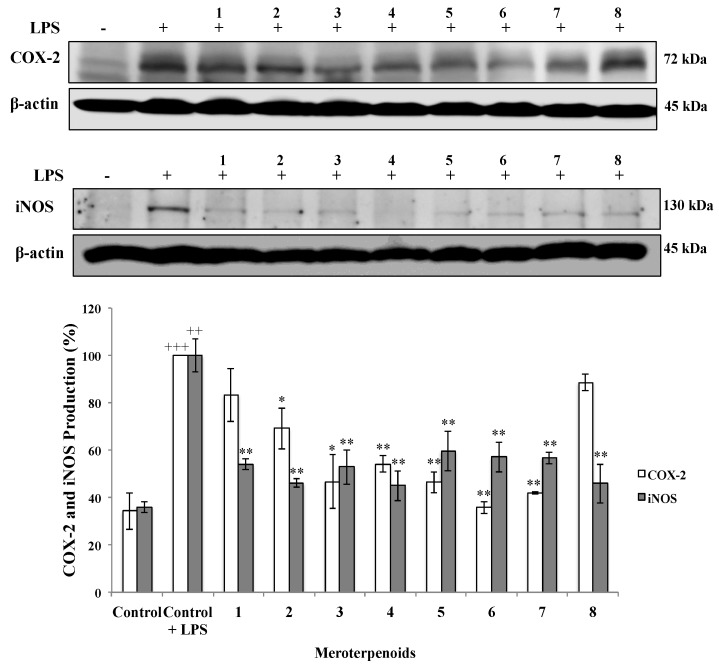
Effect of meroterpenoids **1**–**8** on LPS-induced COX-2 and iNOS protein expression in THP-1 macrophages. Cells were pretreated for 1 h with the compounds (**1**, **2**, **3**, **4**, **7**, and **8** at 8 μg/mL; **5** and **6** at 4 μg/mL) and then stimulated with LPS (1 μg/mL). Cytosolic lysates from 24 h-stimulated cells were separated on 10% SDS-PAGE. COX-2, iNOS, and β-actin were detected by western blot analysis. Data are expressed as means ± SE from three independent experiments. Statistical significance is indicated (+++ *p* < 0.001 and ++ *p* < 0.01, vs. Control, respectively; * *p* < 0.05, and ** *p* < 0.01 vs. Control + LPS).

COX-2 is the key enzyme regulating the production of prostaglandins, which are the central mediators of inflammation. On the other hand, iNOS enzyme represents an important molecular target closely involved in inflammatory responses. Thus, the effect of the AMTs **1**–**8** on LPS-induced COX-2 and iNOS protein expression was investigated by western blot analysis. As shown in Figure 3, the expression of COX-2 and iNOS proteins was markedly augmented in THP-1 macrophages upon LPS treatment. The pretreatment with the AMTs **2**, **3**, **4**, **5**, **6**, and **7** significantly down-regulated the expression of COX-2, while no significant effect was observed for compounds **1** and **8**. The more active compounds were **3**, **5**, **6**, and **7**, which decreased COX-2 levels by 53.35%, 53.52%, 64.23%, and 58.05% respectively. On the other hand, AMTs **1**–**8** effectively suppressed LPS-induced iNOS expression, decreasing iNOS levels in the range 40.31%–54.93%.

### 2.2. Anticancer Activity

#### 2.2.1. Cytotoxic Effects and Selectivity of the AMTs **1**–**8**

**Figure 4:**
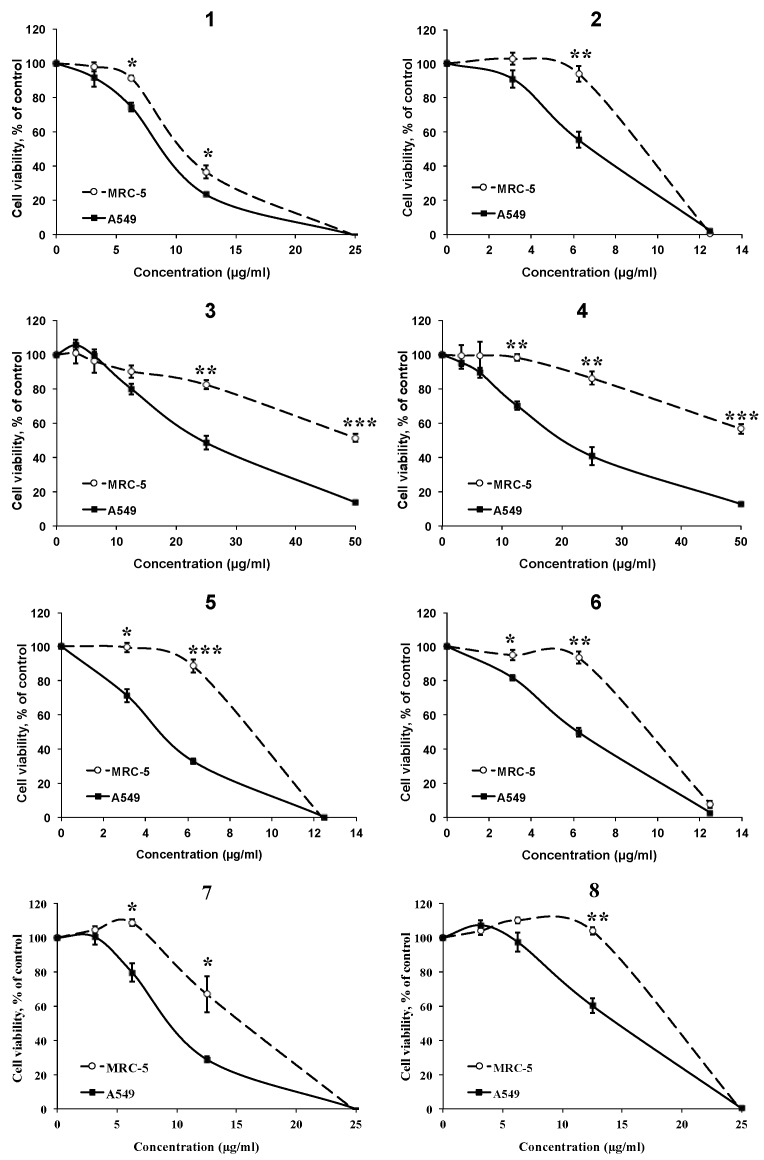
Effects of different concentrations of meroterpenoids **1**–**8** on the viability of human lung cancer cell line A549 and the human fetal lung fibroblastic MRC-5 cells, using MTT assay after 72 h of treatment. Data are mean ±SE of three independent experiments. * *p* < 0.05, ** *p* < 0.01 and *** *p* < 0.01 between the two cell lines.

The effect of the AMTs **1–8** on cell viability was investigated in the human lung cancer cell line A549. The results (Figure 4) indicated that all the AMTs inhibited the A549 cell growth after 72 h of incubation in a dose-dependent manner. The most active compounds were **1**, **2**, **5**, and **6** with IC_50_ values of 8.68, 6.61, 4.56, and 6.19 μg/mL, respectively (Table 1). Since the AMTs **1**–**8** showed an interesting cytotoxic activity towards A549 cells, the compounds were similarly evaluated on a normal cell line (human fetal lung fibroblastic MRC-5 cells) to determine the selectivity index (SI = IC_50_ value for normal cells/IC_50_ value for cancer cells). It has been reported that compounds with SI value higher or equal to 2.0 are potentially selective [34]. According to the data in Table 1, all AMTs showed selective cytotoxicity against the cancer cells; especially compounds **3** and **4**, which at increasing concentrations maintained higher toxicity against A549 cancer cells than against MRC-5 non-malignant cells (Figure 4). It is worth noting that after treatment with 50 μg/mL of these two AMTs, the cell viability was lower than 15% for cancer cells and higher than 55% for normal cells. On the other hand, **1** was the less selective compound; its SI was 1.22.

#### 2.2.2. Effects of AMTs **1**–**8** on A549 Cell Cycle Progression

**Figure 5:**
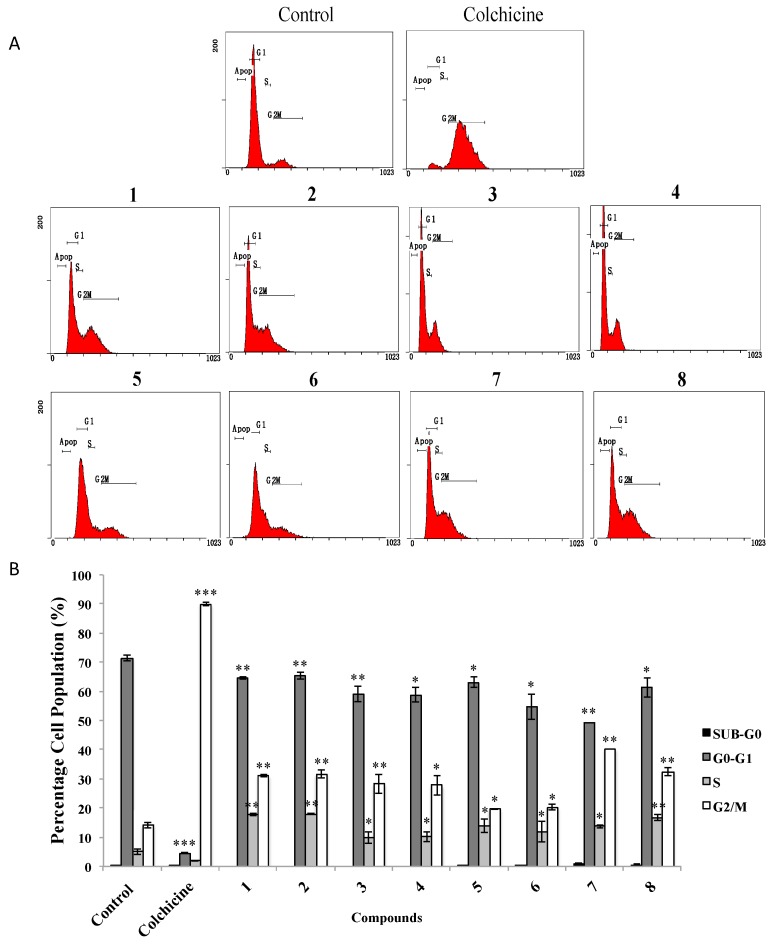
Effects of meroterpenoids **1**–**8** on cell cycle distribution of A549 cells. Cells were incubated with IC_50_ doses of the compounds for 24 h. (**A**) Cells were harvested to measure the cell cycle distribution by flow cytometry. (**B**) Quantitative analysis of cell cycle distribution after treatment. Data represent mean ±SE from three independent experiments. * *p* < 0.05, ** *p* < 0.01, and *** *p* < 0.001 compared with the untreated group (control).

We next investigated whether the treatment with AMTs **1**–**8** caused any cell cycle-related event, which inhibit the viability of A549 cell line. Thus, the cells were treated for 24 h with the AMTs at concentrations of IC_50_ (Table 1), or with colchicine (at 0.2 μg/mL) as the positive control, and then subjected to flow cytometry analysis to evaluate the distribution of cells in the different phases of the cell cycle (Figure 5A). The results showed that most of the AMTs assayed induced a significant accumulation of cells at the G2/M phase (20.20%–40.19%) and at the S phase (9.73%–17.91%) of the cell cycle, with a parallel depletion of the percentage of cells in G0/G1 phase (49.31%–65.43%), while colchicine arrested 89.88% of cells in G2/M phase. The AMTs **1**, **2**, **7**, and **8** were the most active and caused a remarkably increase of cells at G2/M phase (31.15%, 31.62%, 40.19%, and 32.50%, *p* < 0.01, respectively) compared with the control group (14.06%), and at S phase (17.75%, 17.90%, 13.69%, and 16.73%, *p* < 0.01, respectively) compared with the control group (4.99%). In parallel, a decrease of the population at G0/G1 phase was observed in the cells treated with these compounds (64.52%, 65.42%, 49.31%, *p* < 0.01 and 61.29%, *p* < 0.05, respectively) in comparison with the control group (71.40%) (Figure 5B).

## 3. Discussion

In the present study, we have expanded the knowledge about the in vitro anti-inflammatory and anticancer activities of eight AMTs (**1**–**8**) isolated from the brown alga *C. usneoides* [33].

First, we investigated the anti-inflammatory effects of the AMTs **1**–**8** and their molecular mechanisms in LPS-induced THP-1 macrophages. The production of pro-inflammatory mediators including TNF-α, IL-6, and IL-1β by macrophages exposed to endotoxins is well established [35,36]. TNF-α plays a major role in initiating and regulating the release of adhesion molecules and the expression of inflammatory mediators during inflammatory responses [37]. IL-6 is a multifunctional cytokine that plays a role in inflammatory responses through the stimulation of acute phase responses, hematopoiesis, and immune reactions [38]. Moreover, inhibition of IL-6 signaling has been successfully translated into the clinic as a powerful anti-inflammatory strategy [39]. IL-1β is one of the most potent pro-inflammatory cytokines, which affects a large number of cellular responses and mediates inflammatory processes at local and systemic levels [40,41]. These pro-inflammatory mediators can induce cell and tissue damage and also activate macrophages in various inflammation-associated diseases [42]. The protein COX-2 is an important inflammatory enzyme responsible for the high prostaglandin levels widely observed in inflammatory pathology [43]. The enzyme iNOS is greatly expressed in macrophages and its activation leads to organ destruction in some inflammatory and autoimmune diseases [44]. Therefore, treatments aimed to suppressing pro-inflammatory cytokines and enzymes are regarded an effective therapeutic strategy for the control of several disorders, including inflammatory diseases.

In this study, we found that the AMTs **1**–**8** significantly reduced the secretion of the pro-inflammatory cytokines TNF-α, IL-6, and IL-1β, as well as inhibited the expressions of proteins COX-2 and iNOS in LPS-induced THP-1 macrophages. These findings are in line with previous results from our group showing that other related AMTs reduce TNF-α expression in LPS-stimulated THP-1 cells [45]. Moreover, the results obtained for the AMTs **1**–**8** represent the first account on the activity of this class of meroterpenoids as inhibitors of the pro-inflammatory mediators IL-6, IL-1ß, COX-2 and iNOS. Overall, the AMTs **1** and **2** were the most potent inhibitors of the production of the three pro-inflammatory cytokines, causing decreases in the range 71.20%–84.43% at 8 μg/mL; at this concentration, compound **2** also significantly inhibited COX-2 and iNOS. The inhibitory effects observed for this molecule (compound 2, also 11-hydroxy-1′-*O*-methylamentadione or AMT-E) on THP-1 cells are consistent with the in vivo study described in our previous report, which showed exerting intestinal anti-inflammatory activity in colitis by down-regulating TNF-α, IL-1ß, and IL-10, as well as suppressing COX-2 and iNOS expression in the mouse colon tissue [46].

Recent reports have also demonstrated the anti-inflammatory potential of a variety of algal terpenes and meroterpenes [23]. A number of compounds have been assayed on LPS-stimulated RAW264.7 macrophages [47,48,49]. Thus, the diterpenoid nerogioltriol from the red alga *Laurencia glandulifera* was found to inhibit the activation of NF𝜅B and the production of NO, TNF-𝛼, and COX-2 [47], while the sesquiterpene 5β-hydroxypalisadin B from *L. snackeyi* [48] and the meroterpene sargachromanol G from *Sargassum siliquastrum* [49] were shown to inhibit the production of NO, TNF-𝛼, IL-6, and IL-1β, as well as to reduce the COX-2 and iNOS expression in LPS-stimulated RAW264.7 macrophages. Other terpenes from *Dictyota plectens* showed anti-inflammatory effects by inhibiting the LPS-induced NO production in mouse peritoneal macrophages [50]. Recently, several halogenated sesquiterpenoids from the red alga *Laurencia tristicha* and meroterpenoids from the brown alga *Homoeostrichus formosana*, also showed an interesting anti-inflammatory ability by inhibiting the *N*-formylmethionyl-leucyl-phenylalanine cytochalasin B (fMLP/CB)-induced superoxide anion (O_2_^−•^) generation and elastase release in human neutrophils [51,52]. The results obtained in our study of the effects of the AMTs **1**–**8** on LPS-stimulated THP-1 macrophages are in line with the anti-inflammatory activities described above. Because these inflammatory mediators have an important role during carcinogenesis and they are secreted by M1 macrophages and other immune cells, terpenoids from *C. usneoides* may have the potential to prevent carcinogenesis. Further research is needed to elucidate the anti-inflammatory potential of these compounds.

We next investigated the effects of the AMTs 1–8 on the viability of the lung cancer cells A549. It is well known that chronic inflammation is associated with several chronic diseases including cancer [53,54]. Cancer is the second leading cause of death worldwide, with lung cancer recognized as one of the most mortal cancer types (OMS, 2018). The metastatic cancer is an incurable disease for most patients because the current anticancer therapies lack enough selective cytotoxicity to kill cancer cells without affecting healthy tissues [55]. Therefore, the development of new drugs with higher selectivity towards cancer cells is vital to advance towards the cure to this deadly disease. Nature has provided useful anticancer drugs (e.g., the vinca alkaloids and the diterpene taxol)—and it is still a source of new agents [56]. In this line, along the last decades, an array of new anticancer compounds has also been isolated from marine organisms [57].

In this study, we have shown the anticancer activity of eight AMTs obtained from the alga *C. usneoides*. In particular, we studied the cytotoxicity of the AMTs **1**–**8** against lung cancer cells and lung normal cells. All AMTs significantly inhibited the survival of A549 cancer cells. Interestingly, the compounds exhibiting a chain of 20 carbon atoms (**1**, **2**, **5**–**8**) were more cytotoxic (IC_50_ ranging from 4.56 to 13.96 μg/mL) than those with a chain of 14 carbon atoms (**3** and **4**). Moreover, we found that the cancer cells were more sensitive to the cytotoxic effect of AMTs **1**–**8** than MRC-5 normal cells. Compounds **3** and **4** had the highest selectivity towards cancer cells, showing selective cytotoxic activity at several concentrations assayed (from 12 to 50 μg/mL). Among algae-derived metabolites, a few terpenoids and meroterpenoids have also been recently reported to possess anticancer activity against lung cancer cells A549; thus the sesquiterpenes elatol from *Laurencia microcladia* together with 5β-hydroxyaplysin and its hydroperoxy analogue from *L. okamurai* had cytotoxic effects towards A549 human cancer cell line with IC_50_ values of 4.8, 35.3, and 15.4 μM, respectively [58,59], while the diterpenes sphaerococcenol A, and two related analogues from *Sphaaerococcus coronopifolius*, inhibited the growth of A549 cells with IC_50_ values of 3.7, 19.0, and 18.0 μM, respectively [60]. Likewise, meroditerpenoids from algae of the genus *Callophycus* exhibited cytotoxicity towards several human cancer cell lines, including A549 cells, with the higher activity observed for bromophycolides M, N, O, P, Q and bromophycoic acid D (mean IC_50_ values of 3.1, 8.6, 9.7, 7.9, 2.0, and 6.8 μM, respectively) [61,62]. In view of these data from the literature, the activity of AMTs **1**, **2**, and **5**–**8** against A549 cells is comparable with several of the values described above.

We have also demonstrated that most of the AMTs suppress the proliferation of A549 by arresting the cell cycle progression at the G2/M and S phases. The cell cycle checkpoints play a key role in the machinery that controls cell division by sensing defects occurring in vital processes, such as DNA replication or chromosome segregation, inducing a cell cycle arrest until the repair of the detected defects [63]. The effect of algal terpenes on lung cancer and cell cycle distribution has been scarcely documented, and less in A549 cells. A report on the sesquiterpene elatol, isolated from *Laurencia microcladia* [58], showed anticancer properties by inducing cell cycle arrest in the G1 and the sub-G1 phases in several human cancer cell lines, including A549 cell line. Our results suggest, for the first time, that the meroterpenoids **1–8** affect the molecular pathways that control the A549 cell cycle progression by arresting the cells at the G2/M and S checkpoints.

## 4. Materials and Methods 

### 4.1. Isolation and Characterization of Meroterpenoids ***1**–**8***

The collection of the alga samples, preparation of the extract, purification and structural characterization of the meroterpenoids **1**–**8** was performed, as previously described [33]. Briefly, shade-dried samples of *C. usneoides* collected at the Gibraltar Strait were ground and extracted with acetone/methanol (MeOH). The resulting extract was subjected to column chromatography (CC) on silica gel (Merck KGaA, Darmstadt, Germany) eluting with *n*-hexane/diethyl ether (Et_2_O) mixtures of increasing polarity, then Et_2_O, chloroform/MeOH mixtures, and finally MeOH. The fractions eluted with *n*-hexane /Et_2_O (30:70, *v*/*v*), Et_2_O, and chloroform/MeOH (95:5, *v*/*v*) were subjected to CC on silica gel (Merck) using *n*-hexane/ethyl acetate (EtOAc) mixtures as eluents. Further separation of selected subfractions by normal phase HPLC using *n*-hexane/EtOAc (60:40 and 50:50, *v*/*v*) or *n*-hexane/isopropanol (90/10, *v*/*v*) as eluents led to obtain compounds **1**–**8**. HPLC separations were performed on a LaChrom-Hitachi apparatus (Merck), equipped with LiChrospher Si-60 (250 × 10 mm, 10 μm) (Merck) and Luna Si (2) (250 × 4.6 mm, 5 μm) (Phenomenex, Torrance, CA, USA) columns, using an RI-71 differential refractometer or L-7400 UV detector (Merck). The isolated compounds were identified by using nuclear magnetic resonance (NMR) and mass spectrometry (MS). NMR spectra were recorded on an Agilent 500 spectrometer (Agilent Technologies, Santa Clara, CA, USA) using CD_3_OD or CDCl_3_ (Sigma-Aldrich, St. Louis, MO, USA) as solvent. MS spectra were obtained on a Waters SYNAPT G2 spectrometer (Waters, Milford, MA, USA). Fully assigned spectroscopic data of the isolated compounds can be found in our previous paper [33].

### 4.2. Reagents for Anti-Inflammatory and Anticancer Assays

Sulforhodamine B (SRB), 3-(4,5-dimethylthiazol-2-yl)-2,5-diphenyl-tetrazolium bromide salt (MTT), dimethylsulfoxide (DMSO), Propidium Iodide (PI), Tris-base, acetic acid, trichloroacetic acid (TCA), colchicine and RNase were from Sigma-Aldrich (Munich, Germany); RPMI 1640 medium and fetal bovine serum (FBS) were from GIBCO (Grand Island, NY, USA); phosphate buffer saline (PBS), streptomycine, penicillin, and trypsine-EDTA were from PAA Laboratories (Pasching, Austria). Phorbol myristate acetate (PMA) was from Sigma-Aldrich Química (Madrid, Spain). For western blotting, anti-COX-2 (Cayman Chemical^®^, Ann Arbor, MI, USA), anti-iNOS (Stressgen-Enzo Life Sciences, Farmingdale, NY, USA), anti-rabbit IgG antibody (Dako^®^ Cytomation, Carpinteria, CA, USA), anti-β-actin (Santa Cruz Biotechnology, Dallas, TX, USA) were purchased.

### 4.3. Anti-Inflammatory Assays

#### 4.3.1. Cell Culture

THP-1 human monocytic leukemia cell line was obtained from the American Type Culture Collection (TIB-202, ATCC, Manassas, VA, USA). The cells were cultured in RPMI 1640 medium containing 10% heat-inactivated FBS, 100 U/mL penicillin, and 100 mg/mL streptomycin, at 37 °C in humidified air containing 5% CO_2_.

#### 4.3.2. Cell Viability Assay

The viability of THP-1 cells was measured by the SRB assay [64]. The cells were seeded in 96-well plates with the growth medium at a density of 1 × 10^4^ cells per well, and differentiation into macrophages was induced by 0.2 μM of phorbol myristate acetate (PMA) [65]. Three days after differentiation into macrophages, the cells were treated with various concentrations (0, 3.125, 6.25, 12.5, 25, 50, 100 µg/mL) of the AMTs **1**–**8** in fresh medium and incubated for another 72 h. Then, the cells were fixed with 50 μL of TCA (50%) and processed, as described in the literature [64].

#### 4.3.3. Determination of Pro-Inflammatory Cytokines Production

THP-1 cells were plated at a density of 3 × 10^5^ cells/mL in 24-well plates and incubated with PMA (0.2 μM) for 72 h in a humidified atmosphere of 5% CO_2_ at 37 °C. The macrophages were pre-treated for 1 h with AMTs **1**–**8** (8 μg/mL for compounds **1**, **2**, **3**, **4**, **7**, and **8**, and 4 μg/mL for **5** and **6**) and then stimulated with lipopolysaccharide (LPS, 1 μg/mL) for another 24 h. Dexamethasone (Dex) was used as positive reference compound at 1 μM. The viability of cells was greater than 95% throughout the experiment. The levels of TNF-α, IL-6, and IL-1ß in supernatants were measured with enzyme-linked immune-sorbent assay (ELISA) kits (Diaclone GEN-PROBE, Besançon cedex, France) according to the manufacturer’s protocols. The absorbance was determined at 450 nm using a microplate reader. To calculate the concentration of cytokines, a standard curve was constructed using serial dilutions of cytokine standards provided with the kit.

#### 4.3.4. Western Blotting Analysis

Western blotting was used to measure the protein levels of COX-2 and iNOS [65]. THP-1 macrophages were plated at a density of 1 × 10^6^ cells/mL in six-well plates, treated for 1 h with compounds **1**–**8** (8 μg/mL for compounds **1**, **2**, **3**, **4**, **7**, and **8**, and 4 μg/mL for compounds **5** and **6**), and then stimulated with 1 μg/mL of LPS in medium at 37 °C. After 24 h, the cells were washed with ice-cold PBS, collected, suspended in the lysis buffer (250 mM NaCl, 50 mM Tris (pH 7.5), 0.5 mM EDTA, 5 mM EGTA, 8 mM MgCl2, 1 mM PMSF, 0.01 mg/mL pepstatin A, 0.01 mg/mL leupeptin, 0.01 mg/mL aprotinin, 1% Triton X-100) and centrifuged at 12,000× *g* at 4 °C for 3 min to yield cell lysates. Proteins concentration in cell lysates was determined by Bio-Rad Protein Assay, based on the method of Bradford (BioRad, Richmond CA, USA) [66]. Cytosolic proteins (50 μg) were separated with 10% SDS-polyacrylamide gel electrophoresis and transferred on PVDF membranes. The membranes were then blocked with 5% (w/v) non-fat dry milk in Tris-buffered saline containing 0.1% Tween-20 (pH 7.4) (TBST) buffer at room temperature for 1 h. The membranes were washed three times (10 min) in TBST buffer and incubated with specific primary antibodies anti-COX-2 (1:3000) or anti-iNOS (1:1000) diluted in 5% (w/v) non-fat dry milk in TBST buffer, at 4 °C overnight. Then, the membranes were incubated with peroxidase-conjugated bovine peroxidase-conjugated goat anti-rabbit IgG (1: 1000) for 1 h at room temperature. To ascertain that blots were loaded with equal amounts of protein lysates, they were also incubated in the presence of the antibody against β-actin protein (1:10,000). After washing the membrane again with TBST buffer (10 min) three times, the antibody was visualized using an enhanced chemiluminescence light-detecting kit (Super-Signal West Pico Chemiluminescent Substrate, Pierce, IL, USA), according to the manufacturer’s instructions and exposed to an X-ray film (GE Healthcare Ltd., Amersham, UK). The protein band densities were quantified using a Scientific Imaging Systems (Biophotonics ImageJ Analysis Software, National Institute of Mental Health, Bethesda, MD, USA).

### 4.4. Anticancer Assays

#### 4.4.1. Cell Line and Cell Culture 

The human fetal lung fibroblastic MRC-5 cell line and the human lung adenocarcinoma A549 cell line were obtained from European Collection of Cell Cultures and maintained in Dulbecco’s Modified Eagle’s Medium (DMEM) supplemented with 2 mM glutamine, 100 U/mL penicillin, 100 µg/mL streptomycin and 10% FBS. Cell lines were cultured at 37 °C in a humidified atmosphere containing 5% CO_2_.

#### 4.4.2. Cytotoxicity Test

Cell proliferation was evaluated by a modified MTT assay, which measures the mitochondrial dehydrogenase activity [67]. A total of 5 × 10^3^ cells/well (MRC-5 cells) and 3 × 10^3^ cells/well (A549 cells) were cultured in a 96-well plate for 24 h. Then, treatments were added to the cell culture. After 72 h of incubation, the medium was removed and 125 µL of MTT (1 mg/mL in medium) was added to each well and incubated for 4 h. Next, 80 µL of 20% sodium dodecyl sulphate (SDS) was added and incubated for 5 h at 37 °C. The optical density of each well was measured at 540 nm (Synergy HT multiwell plate spectrophotometer reader, BioTek Instruments Inc., Winooski, VT, USA) to quantify cell viability. Cell viability (%) was calculated according to the following formula: % viability = (absorbance of compounds treated cells/absorbance of control cells) × 100. The degree of selectivity of the compounds was expressed by its SI value. Each SI value was calculated using the formula: SI = IC_50_ normal cell/IC_50_ cancer cell.

#### 4.4.3. Cell Cycle Analysis

For cell cycle analysis by flow cytometry, A549 cells were seeded at 1 × 10^6^ cells/well in 6-well plates and incubated for 24 h followed by treatment with the AMTs, at concentration of IC_50_, and further incubation for 24 h. Colchicine (final concentration of 0.2 μg/mL) was used as positive control. Cells were harvested after trypsinization and washed once with PBS. Then, the cells were centrifuged at 1500 rpm for 5 min (25 °C), the pellet was fixed with 1 mL of ice-cold 70% ethanol, and the samples were stored at −4 °C overnight. Then the cells were washed with PBS and incubated in the darkness with PBS containing 5 mg/mL of RNase A for 48 h at 4 °C. Subsequently, 50 µL of propidium iodide (0.1 mg/mL) was added and the cells were incubated for 1 h at 4 °C. The relative DNA content per cell was analyzed using a Beckman Coulter Cytomics FC 500 MPL (Beckman Coulter Inc, San Diego, CA, USA). The data acquisition was performed with the DML program. The analysis of the acquired data was performed with the CXP cytometer.

#### 4.4.4. Statistical Analysis

The results are presented as the mean ± Standard Error (SE) of at least three independent experiments. Data were evaluated with GraphPad Prism^®^ Version 5.00 software (San Diego, CA, USA). Differences between two groups were analyzed by the Student’s *t*-test. Difference with *p* < 0.05 (*), *p* < 0.01 (**) or *p* < 0.001 (***) were considered statistically significant. The cytotoxic activity of a drug was determined against two cell lines; the statistical analysis was carried out to compare the cytotoxicity of a particular concentration of the compound between both cell lines.

## 5. Conclusions

Various AMTs significantly reduced the secretion of the pro-inflammatory cytokines and inhibited the expressions of proteins COX-2 and iNOS in THP-1 activated macrophages. Likewise, most of the AMTs suppressed the proliferation of A549 lung cancer cells by arresting cell cycle progression at the G2/M and S phases.

Because these inflammatory mediators have an important role during carcinogenesis and they are secreted by M1 macrophages and other immune cells, the present results do allow selecting meroterpenoids from *C. usneoides* for future approaches to in vitro and in vivo models of inhibiting inflammation to prevent carcinogenesis. Upcoming experiments in this field are guaranteed.

## Figures and Tables

**Table 1:** IC_50_ values (μg/mL) of meroterpenoids **1**–**8** against the normal lung cells MRC-5 and the lung cancer cells A549, after 72 h of treatment. SI = IC_50_ value for normal cells/IC_50_ value for cancer cells.

Compound	Cell Lines		Selectivity Index
	MRC-5	A549	SI
**1**	10.64 ± 0.50	8.68 ± 0.15	1.22
**2**	8.65 ± 0.18	6.61 ± 0.38	1.30
**3**	51.91 ± 2.76	24.42 ± 1.71	2.12
**4**	58.38 ± 3.30	21.00 ± 1.79	2.78
**5**	8.39 ± 0.14	4.56 ± 0.21	1.83
**6**	8.87 ± 0.05	6.19 ± 0.25	1.43
**7**	14.46 ± 1.14	9.31 ± 0.33	1.55
**8**	17.83 ± 0.09	13.6 ± 0.55	1.28

Data are means ± SE from three independent experiments.

## References

[1] Pan M.H., Lai C.S., Dushenkov S., Ho C.T. (2009). Modulation of inflammatory genes by dietary flavonoids. J. Agric. Food Chem..

[2] Medzhitov R. (2008). Origin and physiological roles of inflammation. Nature.

[3] Lu H., Ouyang W., Huang C. (2006). Inflammation, a key event in cancer development. Mol. Cancer Res..

[4] Niccolai E., Boem F., Emmi J., Amedei A. (2020). The Link "Cancer and autoimmune diseases" in the light of microbiota: Evidence of a potential culprit. Immunol. Lett..

[5] Bremnes R.M., Al-Shibli K., Donnem T., Sirera R., Al-Saad S., Andersen S., Stenvold H., Camps C., Busund L.T. (2011). The role of tumor-infiltrating immune cells and chronic inflammation at the tumor site on cancer development, progression, and prognosis: emphasis on non-small cell lung cancer. J. Thorac. Oncol..

[6] Balkwill F., Mantovani A. (2001). Inflammation and cancer: back to Virchow?. Lancet.

[7] Francescone R., Hou V., Grivennikov S.I. (2014). Microbiome, inflammation, and cancer. Cancer J..

[8] Najafi M., Hashemi Goradel N., Farhood B., Salehi E., Nashtaei M.S., Khanlarkhani N., Khezri Z., Majidpoor J., Abouzaripour M., Habibi M. (2019). Macrophage polarity in cancer: A review. J. Cell. Biochem..

[9] Pinto M.L., Rios E., Durães C., Ribeiro R., Machado J.C., Mantovani A., Barbosa M.A., Carneiro F., Oliveira M.J. (2019). The Two Faces of Tumor-Associated Macrophages and Their Clinical Significance in Colorectal Cancer. Front. Immunol..

[10] Brown J.M., Recht L., Strober S. (2017). The Promise of Targeting Macrophages in Cancer Therapy. Clin. Cancer Res..

[11] Bray F., Ferlay J., Soerjomataram I., Siegel R.L., Torre L.A., Jemal A. (2018). Global Cancer Statistics 2018: GLOBOCAN estimates of incidence and mortality worldwide for 36 cancers in 185 countries. CA Cancer J. Clin..

[12] Ma Z., Yang Y., Fan C., Han J., Wang D., Di S., Hu W., Liu D., Li X., Reiter R.J. (2016). Melatonin as a potential anticarcinogen for non-small-cell lung cancer. Oncotarget.

[13] Torre L.A., Bray F., Siegel R.L., Ferlay J., Lortet-Tieulent J., Jemal A. (2015). Global Cancer Statistics, 2012. CA Cancer J. Clin..

[14] Cho W.C., Kwan C.K., Yau S., So P.P., Poon P.C., Au J.S. (2011). The role of inflammation in the pathogenesis of lung cancer. Expert Opin. Ther. Tar..

[15] Engels E.A. (2011). Inflammation in the development of lung cancer: Epidemiological evidence. Expert Rev. Anticancer Ther..

[16] Azad N., Rojanasakul Y., Vallyathan V. (2008). Inflammation and lung cancer: roles of reactive oxygen/nitrogen species. J. Toxicol. Environ. Health.

[17] Gray Z., Shi G., Wang X., Hu X. (2018). Macrophage inducible nitric oxide synthase promotes the initiation of lung squamous cell carcinoma by maintaining circulated inflammation. Cell Death Dis..

[18] Talero E., García-Mauriño S., Ávila-Román J., Rodríguez-Luna A., Alcaide A., Motilva V. (2015). Bioactive Compounds Isolated from Microalgae in Chronic Inflammation and Cancer. Mar. Drugs.

[19] Wells M.L., Potin P., Craigie J.S., Raven J.A., Merchant S.S., Helliwell K.E., Smith A.G., Camire M.E., Brawley S.H. (2017). Algae as nutritional and functional food sources: Revisiting our understanding. J. Appl. Phycol..

[20] Pereira L., Pomin V.H. (2012). A review of the nutrient composition of selected edible seaweeds. Seaweed: Ecology, Nutritional Composition and Medicinal Uses.

[21] Barsanti L., Gualtieri P. (2006). Algae and men. Algae: Anatomy, Biochemistry, and Biotechnology.

[22] Sonani R.R., Rastogi R.P., Madamwar D. (2017). Natural antioxidants from algae. Algal Green Chem..

[23] Fernando I.P.S., Nah J.W., Jeon Y.J. (2016). Potential anti-inflammatory natural products from marine algae. Environ. Toxicol. Pharmacol..

[24] Shannon E., Abu-Ghannam N. (2016). Antibacterial derivatives of marine algae: An overview of pharmacological mechanisms and applications. Mar. Drugs.

[25] Ruan B.F., Ge W.W., Lin M.X., Li Q.S. (2018). A Review of the components of seaweeds as potential candidates in cancer therapy. Anticancer Agents Med. Chem..

[26] Hussain E., Wang L., Jiang B., Riaz S., Butt G.Y., Shi D. (2016). A review of the components of brown seaweeds as potential candidates in cancer therapy. RSC Adv..

[27] Guiry M.D. AlgaeBase. World-wide electronic publication. http://www.algaebase.org.

[28] Bruno de Sousa C., Gangadhar K.N., Macridachis J., Pavão M., Morais T.R., Campino L., Varela J., Lago J.H.G. (2017). *Cystoseira* algae (Fucaceae): Update on their chemical entities and biological activities. Tetrahedron: Asymmetry.

[29] Gouveia V., Seca A.M.L., Barreto M.C., Pinto D.C.G.A. (2013). Di- and sesquiterpenoids from *Cystoseira* genus: Structure, intra-molecular transformations and biological activity. Mini-Rev. Med. Chem..

[30] Amico V. (1995). Marine brown algae of family Cystoseiraceae: Chemistry and chemotaxonomy. Phytochemistry.

[31] Valls R., Piovetti L. (1995). The chemistry of the Cystoseiraceae (Fucales: Pheophyceae): Chemotaxonomic relationships. Biochem. Syst. Ecol..

[32] Zbakh H., Zubía E., De Los Reyes C., Calderón-Montaño J.M., Motilva V. (2020). Anticancer activities of meroterpenoids isolated from the brown alga *Cystoseira usneoides* against the human colon cancer cells HT-29. Foods.

[33] De los Reyes C., Zbakh H., Motilva V., Zubía E. (2013). Antioxidant and anti-inflammatory meroterpenoids from the brown alga *Cystoseira usneoides*. J. Nat. Prod..

[34] Suffness M., Pezzuto J.M., Hostettmann K. (1990). Assays related to cancer drug discovery. Methods in Plant Biochemistry: Assays for Bioactivity.

[35] Li W., Wang Y., Wang X., He Z., Liu F., Zhi W., Zhang H., Niu X. (2016). Esculin attenuates endotoxin shock induced by lipopolysaccharide in mouse and NO production in vitro through inhibition of NF-κB activation. Eur. J. Pharmacol..

[36] Rios E.C., Soriano F.G., Olah G., Gerö D., Szczesny B., Szabo C. (2016). Hydrogen sulfide modulates chromatin remodeling and inflammatory mediator production in response to endotoxin, but does not play a role in the development of endotoxin tolerance. J. Inflamm. (Lond).

[37] McCoy M.K., Ruhn K.A., Blesch A., Tansey M.G. (2011). TNF: A key neuroinflammatory mediator of neurotoxicity and neurodegeneration in models of Parkinson’s disease. Adv. Exp. Med. Biol..

[38] Tanaka T., Narazaki M., Kishimoto T. (2014). IL-6 in inflammation, immunity, and disease. Cold Spring Harb. Perspect. Biol..

[39] Baran P., Hansen S., Waetzig G.H., Akbarzadeh M., Lamertz L., Huber H.J., Ahmadian M.R., Moll J.M., Scheller J. (2018). The balance of interleukin (IL)-6, IL-6·soluble IL-6 receptor (sIL-6R), and IL-6·sIL-6R·sgp130 complexes allows simultaneous classic and trans-signaling. J. Biol. Chem..

[40] Dinarello C.A. (2018). Overview of the IL-1 family in innate inflammation and acquired immunity. Immunol. Rev..

[41] Borthwick L.A. (2016). The IL-1 cytokine family and its role in inflammation and fibrosis in the lung. Semin. Immunopathol..

[42] Yoon W.J., Ham Y.M., Kim S.S., Yoo B.S., Moon J.Y., Baik J.S., Lee N.H., Hyun C.G. (2009). Suppression of proinflammatory cytokines, iNOS, and COX-2 expression by brown algae *Sargassum micracanthum* in RAW264.7 macrophages. Eurasia J. Biosci..

[43] Murakami A., Ohigashi H. (2007). Targeting NOX, INOS and COX-2 in inflammatory cells: chemoprevention using food phytochemicals. Int. J. Cancer.

[44] Pansanit A., Park E.J., Kondratyuk T.P., Pezzuto J.M., Lirdpra-pamongkol K., Kittakoop P. (2013). Vermelhotin, an anti-inflammatory agent, suppresses nitric oxide production in RAW 264.7 cells via p38 inhibition. J. Nat. Prod..

[45] De los Reyes C., Ortega M., Zbakh H., Motilva V., Zubía E. (2016). *Cystoseira usneoides*: A brown alga rich in antioxidant and anti-inflammatory meroditerpenoids. J. Nat. Prod..

[46] Zbakh H., Talero E., Avila J., Alcaide A., De los Reyes C., Zubía E., Motilva V. (2016). The Algal Meroterpen 11-Hydroxy-11-O-Methylamentadione Ameloriates Dextran Sulfate Sodium-Induced Colitis in Mice. Mar. Drugs.

[47] Chatter R., Ben Othman R., Rabhi S., Kladi M., Tarhouni S., Vagias C., Roussis V., Guizani-Tabbane L., Kharrat R. (2011). *In vivo* and *in vitro* anti-inflammatory activity of neorogioltriol, a new diterpene extracted from the red algae *Laurencia glandulifera*. Mar. Drugs.

[48] Wijesinghe W.A.J.P., Kang M.C., Lee W.W., Lee H.S., Kamada T., Vairappan C.S., Jeon R.J. (2014). 5β-Hydroxypalisadin B isolated from red alga *Laurencia snackeyi* attenuates inflammatory response in lipopolysaccharide-stimulated RAW 264.7 macrophages. Algae.

[49] Yoon W.J., Heo S.J., Han S.C., Lee H.J., Kang G.J., Kang H.K., Hyun J.W., Koh Y.S., Yoo E.S. (2012). Anti-inflammatory effect of sargachromanol G isolated from *Sargassum siliquastrum* in RAW 264.7 Cells. Arch. Pharm. Res..

[50] Zhao M., Cheng S., Yuan W., Dong J., Huang K., Sun Z., Yan P. (2015). Further new xenicanes from a Chinese collection of the brown alga *Dictyota plectens*. Chem. Pharm. Bull..

[51] Chen J.Y., Huang C.Y., Lin Y.S., Hwang T.L., Wang W.L., Chiou S.F., Sheu J.H. (2016). halogenated sesquiterpenoids from the red alga *Laurencia tristicha* collected in Taiwan. J. Nat. Prod..

[52] Fang H.Y., Chokkalingam U., Chiou S.F., Hwang T.L., Chen S.L., Wang W.L., Sheu J.H. (2015). Bioactive chemical constituents from the brown alga *Homoeostrichus formosana*. Int. J. Mol. Sci..

[53] Gupta S.C., Kunnumakkara A.B., Aggarwal S., Aggarwal B.B. (2018). Inflammation, a Double-Edge Sword for Cancer and Other Age-Related Diseases. Front. Immunol..

[54] Makvandi M., Sellmyer M.A., Mach R.H. (2017). Inflammation and DNA damage: Probing pathways to cancer and neurodegeneration. Drug Discov. Today Technol..

[55] Siegel R.L., Miller K.D., Jemal A. (2018). Cancer statistics, 2018. CA Cancer J. Clin..

[56] Martin-Cordero C., Leon-Gonzalez A.J., Calderon-Montano J.M., Burgos-Moron E., Lopez-Lazaro M. (2012). Pro-oxidant natural products as anticancer agents. Curr. Drug Targets.

[57] Ercolano G., De Cicco P., Ianaro A. (2019). New Drugs from the Sea: Pro-Apoptotic Activity of Sponges and Algae Derived Compounds. Mar. Drugs..

[58] Campos A., Souza C.B., Lhullier C., Falkenberg M., Schenkel E.P., Ribeiro-do-Valle R.M., Siqueira J.M. (2012). Anti-tumour effects of elatol, a marine derivative compound obtained from red algae *Laurencia microcladia*. J. Pharm. Pharmacol..

[59] Yu X.Q., He W.F., Liu D.Q., Feng M.T., Fang Y., Wang B., Feng L.H., Guo Y.W., Mao S.C. (2014). A seco-laurane sesquiterpene and related laurane derivatives from the red alga *Laurencia okamurai* Yamada. Phytochemistry.

[60] Smyrniotopoulos V., Vagias C., Bruyère C., Lamoral-Theys D., Kiss R., Roussis V. (2010). Structure and in vitro antitumor activity evaluation of brominated diterpenes from the red alga *Sphaerococcus coronopifolius*. Bioorg. Med. Chem..

[61] Lane A.L., Stout E.P., Lin A.S., Prudhomme J., Le Roch K., Fairchild C.R., Franzblau S.G., Hay M.E., Aalbersberg W., Kubanek J. (2009). Antimalarial bromophycolides J-Q from the Fijian red alga *Callophycus serratus*. J. Org. Chem..

[62] Teasdale M.E., Shearer T.L., Engel S., Alexander T.S., Fairchild C.R., Prudhomme J., Torres M., Le Roch K., Aalbersberg W., Hay M.E. (2012). Bromophycoic acids: bioactive natural products from a Fijian red alga *Callophycus* sp.. J. Org. Chem..

[63] Malumbres M. (2012). Cell cycle-based therapies move forward. Cancer Cell.

[64] Skehan P., Storeng R., Scudiero D., Monks A., McMahon J., Vistica D., Warren J.T., Bokesch H., Kenney S., Boyd M.R.J. (1990). New colorimetric cytotoxicity assay for anticancer-drug screening. Natl. Cancer Inst..

[65] Ávila-Román J., Talero E., de Los Reyes C., García-Mauriño S., Motilva V. (2018). Microalgae-derived oxylipins decrease inflammatory mediators by regulating the subcellular location of NFκB and PPAR-γ. Pharmacol. Res..

[66] Bradford M. (1976). A Rapid and Sensitive Method for The Quantitation of Microgram Quantities of Protein Utilizing the Principle of Protein-Dye Binding. Anal. Biochem..

[67] Calderón-Montaño J.M., Jiménez-Alonso J.J., Guillén-Mancina E., Burgos-Morón E., López-Lázaro M. (2018). A 30-s exposure to ethanol 20% is cytotoxic to human keratinocytes: possible mechanistic link between alcohol-containing mouthwashes and oral cancer. Clin. Oral. Investig..

